# 2-Cyano-*N*′-[(1*E*)-1-(3,4-di­meth­oxy­phen­yl)ethylidene]acetohydrazide

**DOI:** 10.1107/S2414314626000891

**Published:** 2026-02-05

**Authors:** Meiyazhagan Manvizhi, Srinivasan Senthilkumar, Sivashanmugam Selvanayagam

**Affiliations:** a Department of Chemistry Annamalai University, Annamalainagar Chidambaram 608 002 India; b PG & Research Department of Physics, Government Arts College, Melur 625 106, India; Vienna University of Technology, Austria

**Keywords:** benzohydrazine, inter­molecular hydrogen bonds, Hirshfeld surface analysis, crystal structure

## Abstract

The non-H part of the mol­ecule of the title compound, C_13_H_15_N_3_O_3_, is nearly planar, with the 2-cyano-*N*′-[(1E)-ethyl­idene]acetohydrazide moiety and the dimeth­oxy phenol ring forming a dihedral angle of 2.5 (1)°. Inter­molecular N—H⋯O, C—H⋯O and C—H⋯π inter­actions are mainly responsible for the cohesion within the crystal structure. The inter­molecular inter­actions were qu­anti­fied and analysed using Hirshfeld surface analysis, revealing that H⋯H inter­actions contribute most to the crystal packing (36.9%). The volume of the crystal voids was calculated to be 167.8 Å^3^ (13% of the unit-cell volume).

## Abstract 2

The mol­ecule of the title compound is nearly planar. In the crystal, the packing of mol­ecules is mainly ensured by N—H⋯O hydrogen bonds, which form *R*_2_^2^ (8) graph-set motifs.

## Graphical abstract

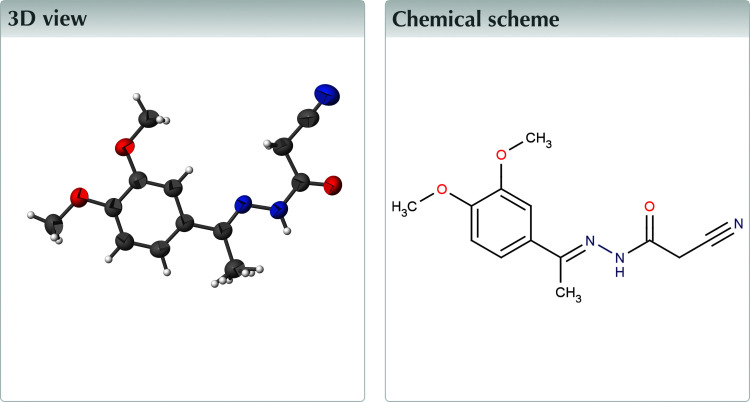

## Structure description

Hydrazone derivatives have long been valued in medicinal and organic chemistry because they are easy to prepare, structurally flexible, and capable of exhibiting a wide range of biological activities. Within this class, acyl hydrazones and heterocycle-linked hydrazones are especially notable, as many of them display anti­diabetic, anti­cancer, anti­microbial, anti­oxidant, and anti-inflammatory properties (Punitha *et al.*, 2020 ▸). In the present work, the title compound, (**I**), was chosen due to the particular ombination of functional groups that are known to enhance biological effectiveness. The hydrazone unit (–C=N–NH–) provides an extended- conjugated system that supports strong inter­molecular inter­actions and can promote favourable binding within enzyme active sites. The 3,4-di­meth­oxy­phenyl ring adds lipophilicity, encouraging π–π stacking and improving membrane permeability, which together may enhance pharmacological performance. The cyano (–C≡N) group, being a strong electron-withdrawing substit­uent, fine-tunes the electronic character of the mol­ecule, increases hydrogen-bond acceptor strength, and is often associated with improved anti­microbial and anti­cancer effects (Senthilkumar *et al.*, 2021 ▸; Maheswari *et al.*, 2025 ▸). The coexistence of electron-donating meth­oxy groups and an electron-withdrawing cyano group creates an inter­nal charge-transfer environment, a feature commonly linked to stronger biological responses in hydrazone frameworks. Because of this combination of structural and electronic attributes, the selected compound offers enhanced biological activity, making it a promising candidate for further pharmacological development (Senthilkumar *et al.*, 2020 ▸).

**Figure 1:**
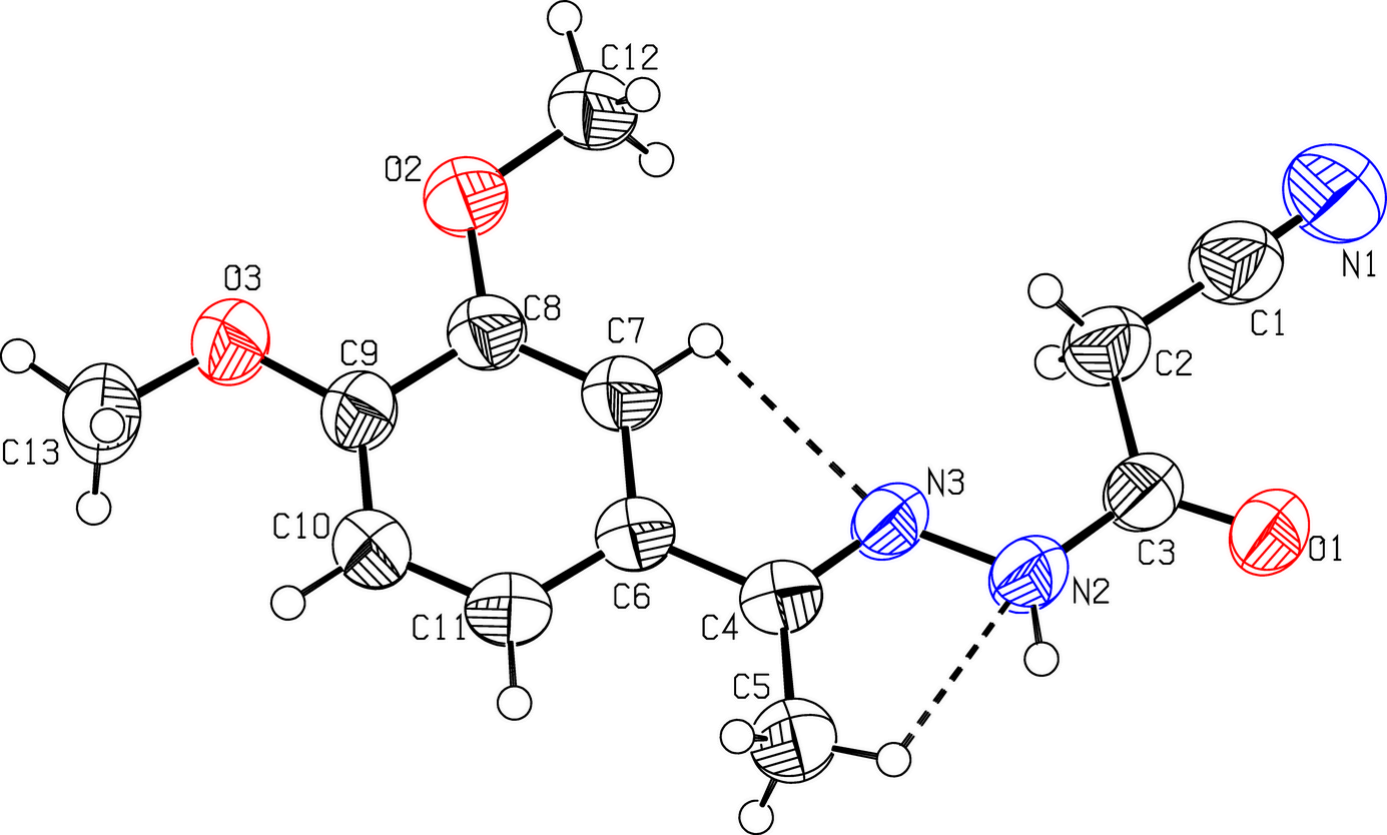
Mol­ecular structure of (**I**) showing the atom-labelling scheme and the intra­molecular hydrogen bonds (dashed lines). Displacement ellipsoids are drawn at the 50% probability level. Only one part of the disordered methyl group at C5 is shown.

The mol­ecular structure of (**I**) is displayed in Fig. 1 ▸. The phenyl ring (C6–C11) is planar with a maximum deviation of 0.010 (3) Å for atom C9, and its attached meth­oxy atoms O2, C12, O3 and C13 deviate by −0.015 (2), 0.075 (4), 0.036 (2) and 0.218 (4) Å, respectively. The 2-cyano-*N*′-[(1*E*)-ethyl­idene]acetohydrazide moiety (N1/C1/C2/C3/O1/N2/N3/C4/C5) is nearly planar with a maximum deviation of 0.120 (4) Å for atom C5. This moiety forms a dihedral angle of 2.5 (1)° with the dimeth­oxy phenyl ring. Weak intra­molecular C5—H5*A*⋯N2 and C7—H7⋯N3 contacts, forming two *S*(5) ring motifs (Bernstein *et al.*, 1995 ▸) may help to establish the solid-state conformation (Table 1 ▸, Fig. 1 ▸).

**Figure 2:**
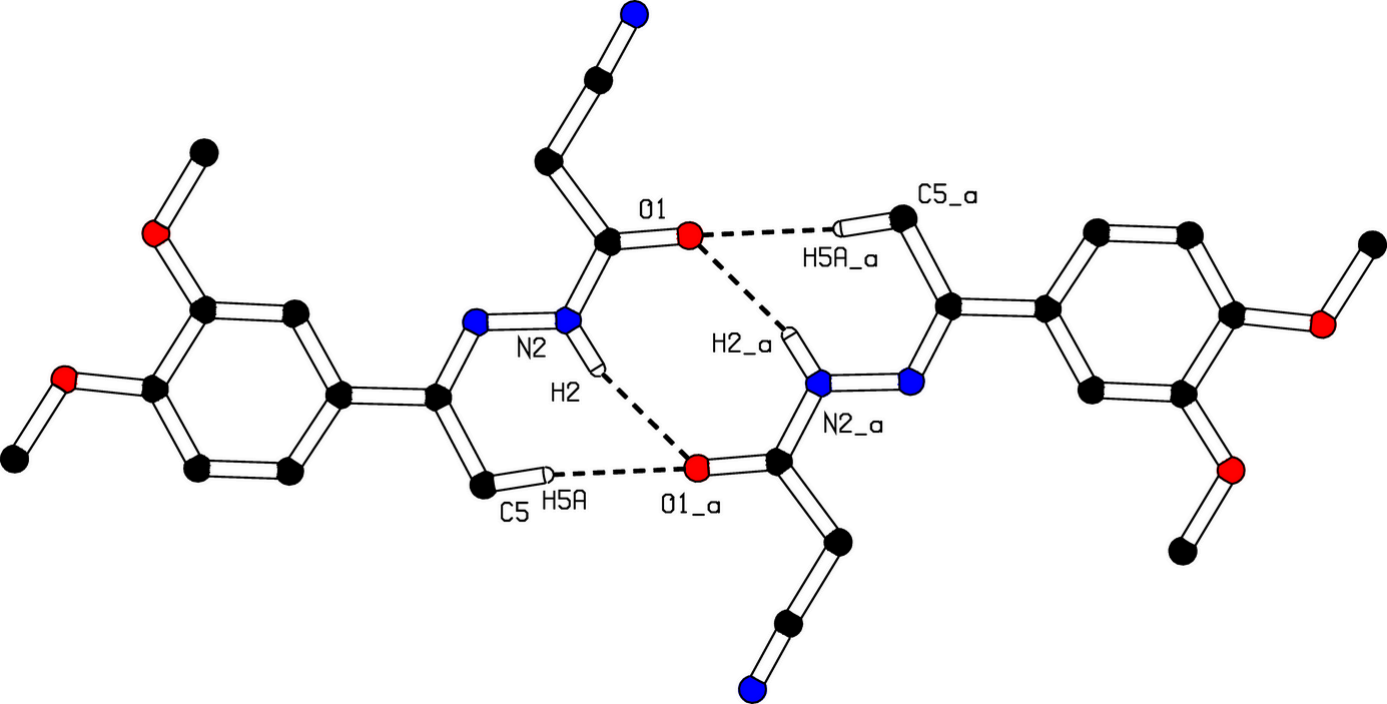
The formation of a centrosymmetric dimer in the crystal structure of (**I**) through N—H⋯O and C—H⋯O hydrogen bonds. [Symmetry code: (*a*) −*x* + 1, −*y* + 1, −*z*].

**Figure 3:**
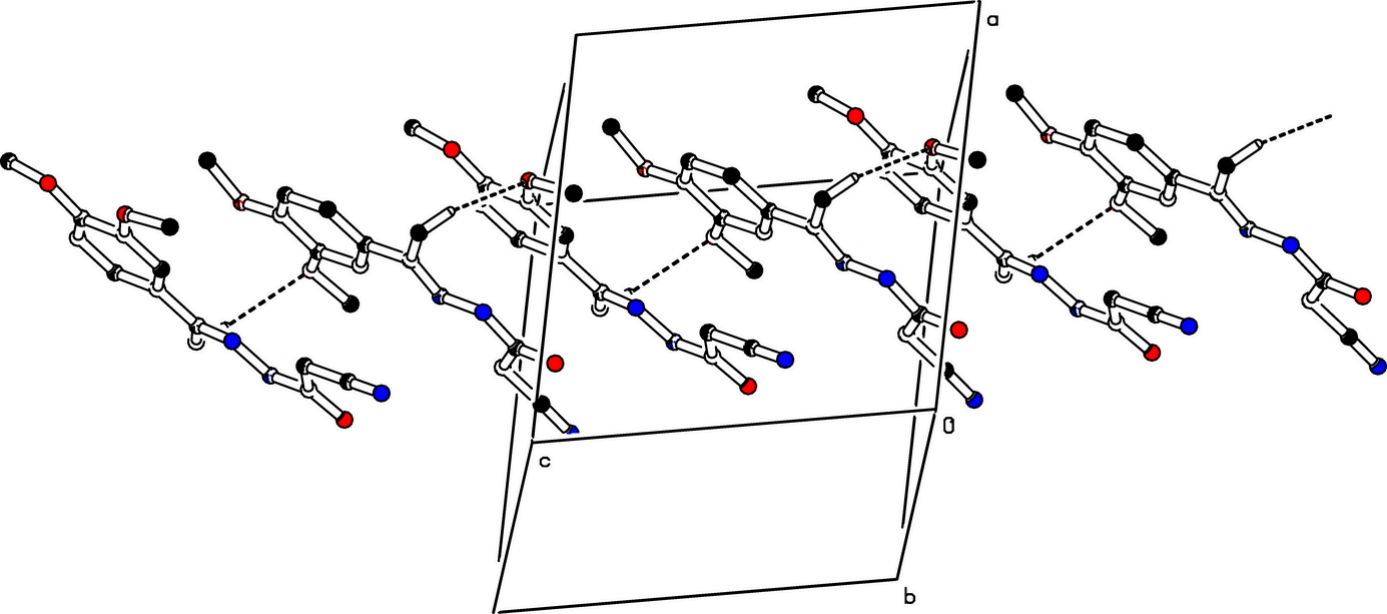
The crystal packing of compound (**I**) viewed along the *b* axis. The C—H⋯O hydrogen bonds are shown as dashed lines. For clarity, H atoms not involved in hydrogen bonds have been omitted.

**Figure 4:**
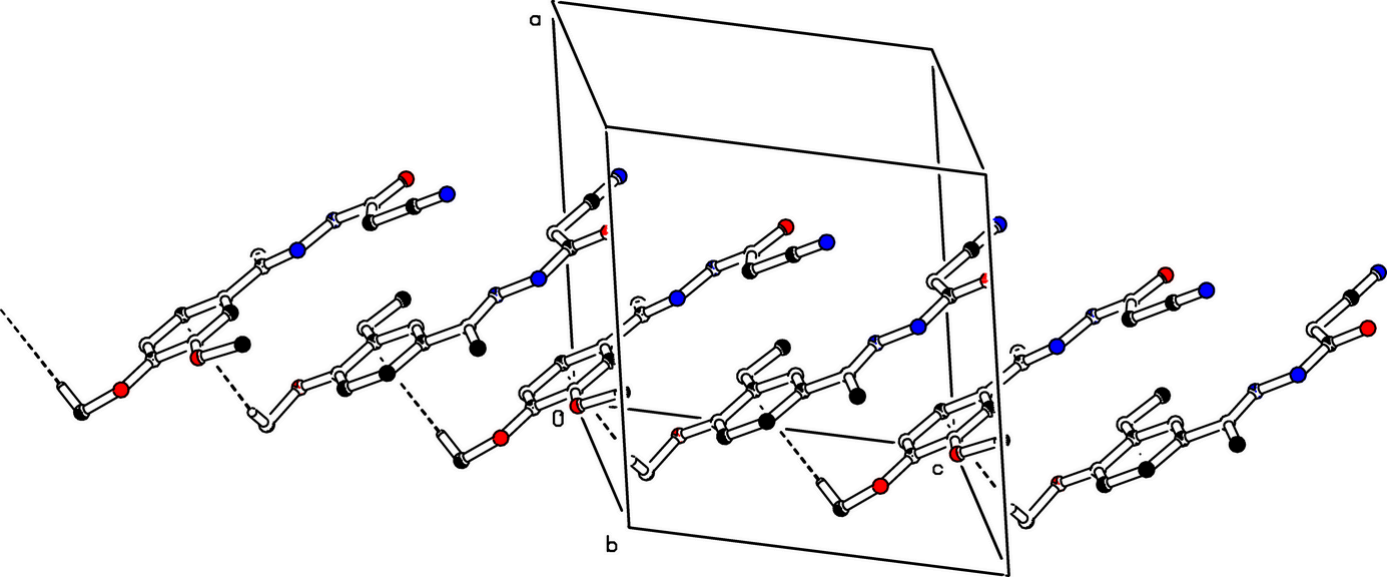
The crystal packing of (**I**). C—H⋯π inter­actions are shown as dashed lines. For clarity, H atoms not involved in these inter­actions have been omitted.

In the crystal, mol­ecules associate pairwise through N2—H2⋯O1^i^ and C5—H5A⋯O1^i^ hydrogen bonds (Table 1 ▸) into inversion dimers with 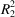
(8) and 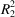
(14) graph-set motifs (Etter *et al.*, 1990 ▸; Bernstein *et al.*, 1995 ▸), as shown in Fig. 2 ▸. The mol­ecules are linked into a *C*(7) chain motif by C5—H5*E*⋯O2^ii^ hydrogen bonds running parallel to [001] (Table 1 ▸, Fig. 3 ▸). Moreover, mol­ecules are further linked along the same direction into a *C*(5) chain motif by C—H⋯π inter­actions, C13—H13B⋯*Cg*, where *Cg* is the centroid of the symmetry-related C6–C11 benzene ring at (*x*, −*y* + 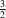, *z* + 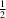) (Table 1 ▸, Fig. 4 ▸).

**Figure 5:**
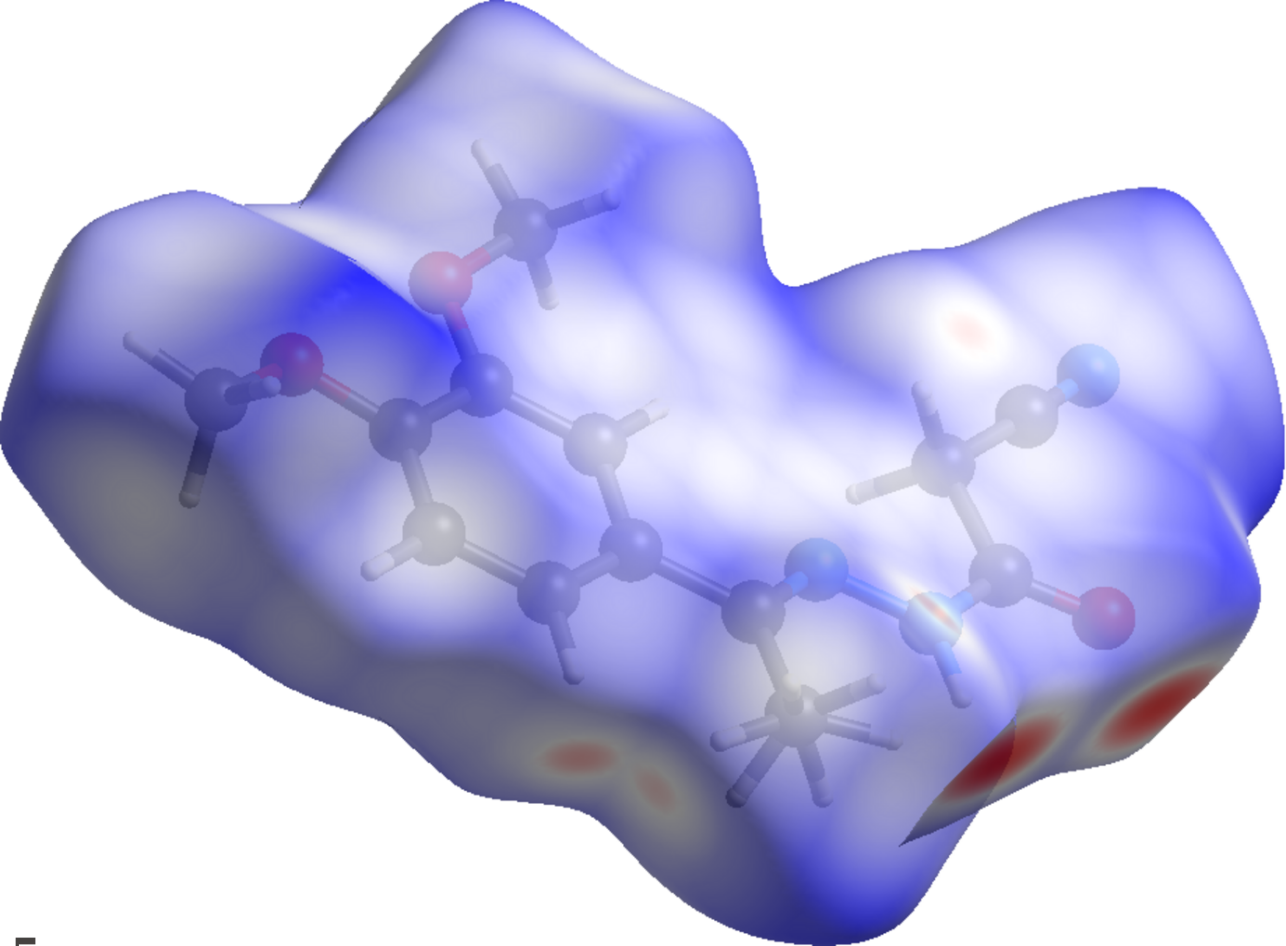
A view of the Hirshfeld surface mapped over *d*_norm_ for compound (**I**).

In order to further characterize and qu­antify the inter­molecular inter­actions in the title compound, a Hirshfeld surface (HS) analysis (Spackman & Jayatilaka, 2009 ▸) was carried out using *CrystalExplorer* (Spackman *et al.*, 2021 ▸). The HS mapped over *d*_norm_ is illustrated in Fig. 5 ▸ where the deep-red spots indicative of strong inter­actions occur at O1, H2 and H5*A*, and these atoms are responsible for inter­molecular N—H⋯O and C—H⋯O hydrogen bonds discussed above.

**Figure 6:**
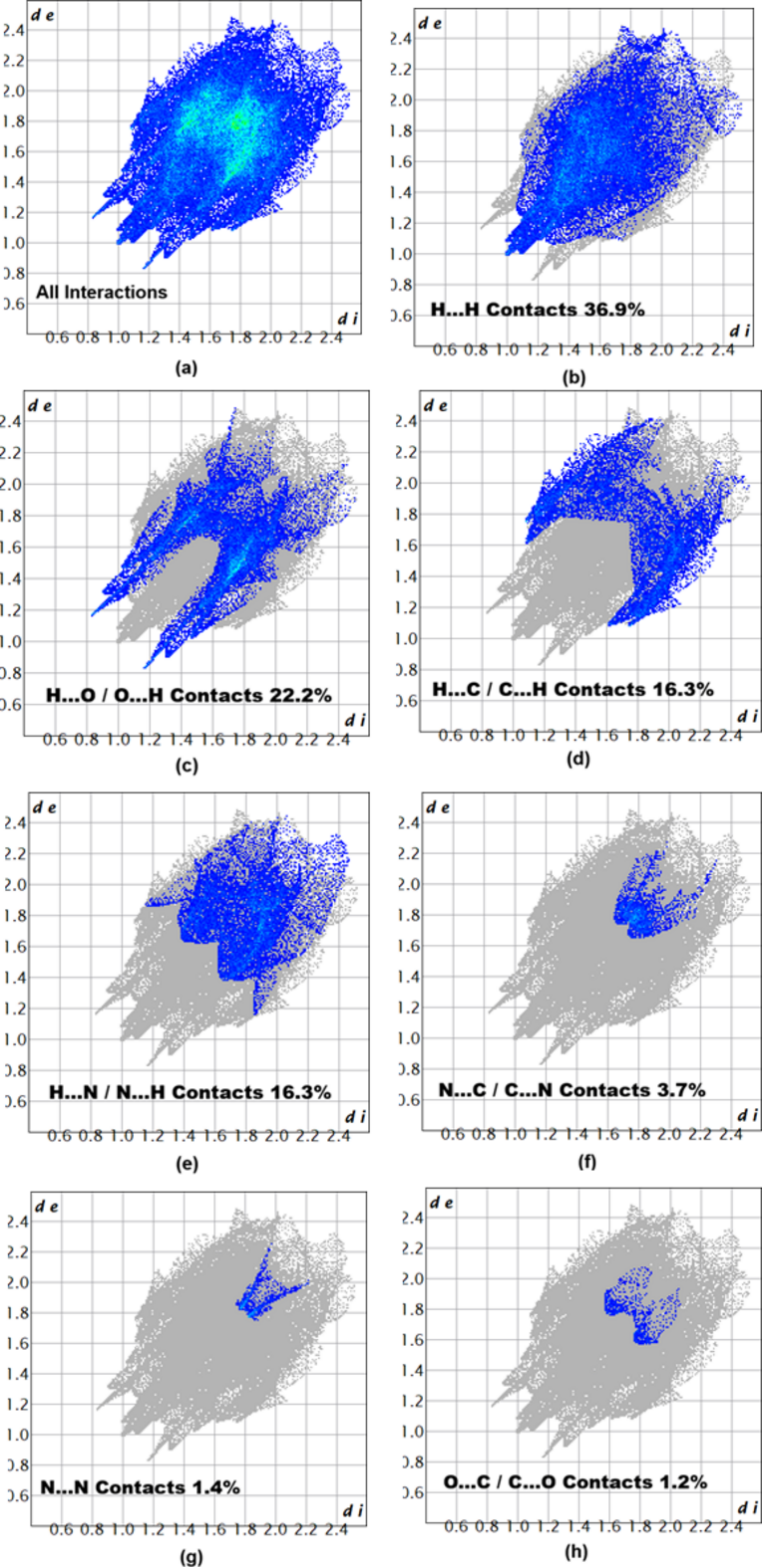
Two-dimensional fingerprint plots for compound (**I**), showing (*a*) all inter­actions, and delineated into (*b*) H⋯H, (*c*) H⋯O/O⋯H, (*d*) H⋯C/C⋯H, (*e*) H⋯N/N⋯H, (*f*) N⋯C/C⋯N, (*g*) N⋯N and (*h*) O⋯C/C⋯O inter­actions. The *d*_i_ and *d*_e_ values are the closest inter­nal and external distances (in Å) from given points on the Hirshfeld surface.

The associated two-dimensional fingerprint plots (McKinnon *et al.*, 2007 ▸) provide qu­anti­tative information about the non-covalent inter­actions in the crystal packing in terms of the percentage contribution of the inter­atomic contacts (Spackman & McKinnon, 2002 ▸). As shown in Fig. 6 ▸, the overall two-dimensional fingerprint plot for compound (**I**) is delineated into the different contact types, revealing that H⋯H (36.9%) and H⋯O/O⋯H (22.2%) are the main contributors to the crystal packing.

**Figure 7:**
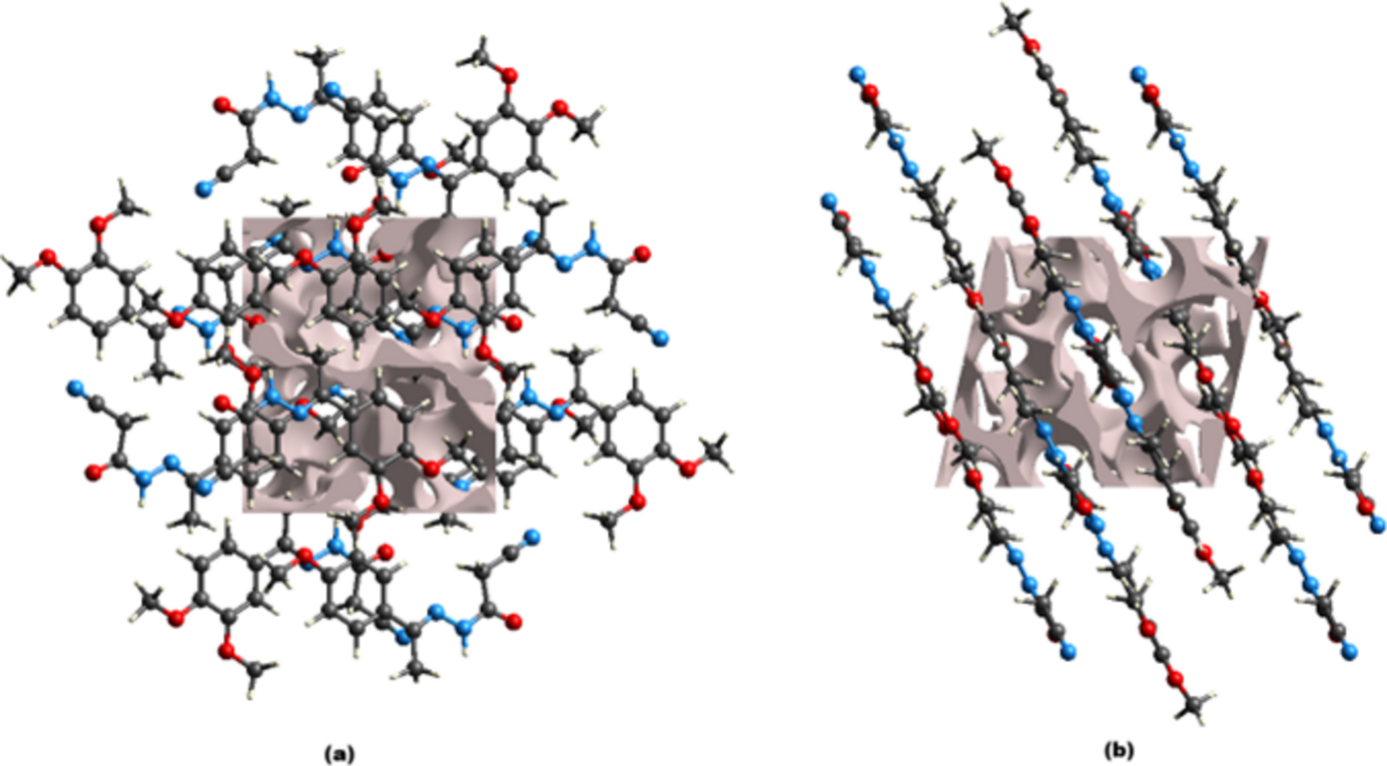
Graphical views of voids in the crystal packing of compound (**I**) viewed down the (*a*) *a-*axis and (*b*) *b*-axis directions.

A void analysis was performed by adding up the electron densities of the spherically symmetric atoms contained in the asymmetric unit (Turner *et al.*, 2011 ▸). The void surface is defined as an isosurface of the procrystal electron density and is calculated for the whole unit cell where the void surface meets the boundary of the unit cell and capping faces are generated to create an enclosed volume. The volume of the crystal voids (Fig. 7 ▸) was calculated to be 168 Å^3^ (13% of the unit-cell volume). Fig. 7 ▸(*b*) also reveals that individual mol­ecules are arranged in layers parallel to (10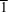).

## Synthesis and crystallization

Compound (**I**) was prepared through a condensation reaction involving an equimolar ratio of 2-cyano­acetohydrazide (0.05 mol) and 3,4-di­meth­oxy­aceto­phenone (0.05 mol). The reagents were placed into a clean reaction flask where methanol served as the reaction medium. A few drops of glacial acetic acid were added to promote the formation of the hydrazone bond. The mixture was then heated under reflux for about 6–8 h. Throughout this period, the progress of the reaction was checked at inter­vals using thin-layer chromatography (TLC) to confirm that the starting materials were being fully consumed. Once the reaction reached completion, the mixture was allowed to cool gradually to room temperature, during which a solid product began to separate out. The resulting precipitate was collected by filtration, thoroughly washed to remove any remaining impurities, and dried under reduced pressure to eliminate traces of solvent. Final purification was achieved by recrystallizing the crude product from warm ethanol solution, affording the desired hydrazone derivative with 75% yield.

## Refinement

Crystal data, data collection and structure refinement details are summarized in Table 2 ▸. The methyl hydrogen atoms at C5 were refined as equally disordered (using an AFIX 127 instruction with *SHELXL*; Sheldrick, 2015*b* ▸) with C—H = 0.98 Å.

## Supplementary Material

Crystal structure: contains datablock(s) I, shelx. DOI: 10.1107/S2414314626000891/wm4242sup1.cif

Structure factors: contains datablock(s) I. DOI: 10.1107/S2414314626000891/wm4242Isup2.hkl

Supporting information file. DOI: 10.1107/S2414314626000891/wm4242Isup3.cml

CCDC reference: 2501701

Additional supporting information:  crystallographic information; 3D view; checkCIF report

## Figures and Tables

**Table 1:** Hydrogen-bond geometry (Å, °) *Cg* is the centroid of the benzene ring (C6–C11).

*D*—H⋯*A*	*D*—H	H⋯*A*	*D*⋯*A*	*D*—H⋯*A*
C5—H5*A*⋯N2	0.96	2.38	2.796 (4)	105
C7—H7⋯N3	0.93	2.44	2.749 (3)	100
N2—H2⋯O1^i^	0.86	2.14	2.982 (3)	166
C5—H5*A*⋯O1^i^	0.96	2.33	3.252 (4)	160
C5—H5*E*⋯O2^ii^	0.96	2.59	3.436 (4)	147
C13—H13*B*⋯*Cg*^iii^	0.97	2.86	3.569 (4)	132

Symmetry codes: (i) 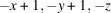; (ii) 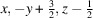; (iii) 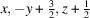.

**Table 2:** Experimental details

Crystal data	
Chemical formula	C_13_H_15_N_3_O_3_
*M*_r_	261.28
Crystal system, space group	Monoclinic, *P*2_1_/*c*
Temperature (K)	300
*a*, *b*, *c* (Å)	11.2308 (13), 11.7792 (13), 10.3668 (12)
β (°)	102.692 (4)
*V* (Å^3^)	1337.9 (3)
*Z*	4
Radiation type	Mo *K*α
μ (mm^−1^)	0.09
Crystal size (mm)	0.46 × 0.12 × 0.06

Data collection	
Diffractometer	Bruker APEXII CCD
Absorption correction	Multi-scan (*SADABS*; Krause *et al.*, 2015 ▸)
*T*_min_, *T*_max_	0.672, 0.746
No. of measured, independent and observed [*I* > 2σ(*I*)] reflections	25559, 3317, 1510
*R*_int_	0.064
(sin θ/λ)_max_ (Å^−1^)	0.667

Refinement	
*R*[*F*^2^ > 2σ(*F*^2^)], *wR*(*F*^2^), *S*	0.067, 0.239, 1.04
No. of reflections	3317
No. of parameters	174
H-atom treatment	H-atom parameters constrained
Δρ_max_, Δρ_min_ (e Å^−3^)	0.34, −0.18

Computer programs: *APEX3* and *SAINT* (Bruker, 2017 ▸), *SHELXT* (Sheldrick, 2015*a* ▸), *ORTEP-3 for Windows* (Farrugia, 2012 ▸), *PLATON* (Spek, 2020 ▸) and *SHELXL* (Sheldrick, 2015*b* ▸).

## full crystallographic data

### 2-Cyano-N'-[(1E)-1-(3,4-dimethoxyphenyl)ethylidene]acetohydrazide Crystal data

**Table:**

C_13_H_15_N_3_O_3_	*F*(000) = 552
*M**_r_* = 261.28	*D*_x_ = 1.297 Mg m^−^^3^
Monoclinic, *P*2_1_/*c*	Mo *K*α radiation, λ = 0.71073 Å
*a* = 11.2308 (13) Å	Cell parameters from 5190 reflections
*b* = 11.7792 (13) Å	θ = 2.5–24.4°
*c* = 10.3668 (12) Å	µ = 0.09 mm^−^^1^
β = 102.692 (4)°	*T* = 300 K
*V* = 1337.9 (3) Å^3^	Plate, colourless
*Z* = 4	0.46 × 0.12 × 0.06 mm

### 2-Cyano-N'-[(1E)-1-(3,4-dimethoxyphenyl)ethylidene]acetohydrazide Data collection

**Table:**

Bruker APEXII CCD diffractometer	1510 reflections with *I* > 2σ(*I*)
Radiation source: i-mu-s microfocus source	*R*_int_ = 0.064
φ and ω scans	θ_max_ = 28.3°, θ_min_ = 1.9°
Absorption correction: multi-scan (SADABS; Krause *et al.*, 2015)	*h* = −14→14
*T*_min_ = 0.672, *T*_max_ = 0.746	*k* = −15→15
25559 measured reflections	*l* = −13→13
3317 independent reflections	

### 2-Cyano-N'-[(1E)-1-(3,4-dimethoxyphenyl)ethylidene]acetohydrazide Refinement

**Table:**

Refinement on *F*^2^	Hydrogen site location: inferred from neighbouring sites
Least-squares matrix: full	H-atom parameters constrained
*R*[*F*^2^ > 2σ(*F*^2^)] = 0.067	*w* = 1/[σ^2^(*F*_o_^2^) + (0.1011*P*)^2^ + 0.4583*P*] where *P* = (*F*_o_^2^ + 2*F*_c_^2^)/3
*wR*(*F*^2^) = 0.239	(Δ/σ)_max_ < 0.001
*S* = 1.04	Δρ_max_ = 0.34 e Å^−^^3^
3317 reflections	Δρ_min_ = −0.18 e Å^−^^3^
174 parameters	Extinction correction: SHELXL (Sheldrick, 2015b), Fc^*^=kFc[1+0.001xFc^2^λ^3^/sin(2θ)]^-1/4^
0 restraints	Extinction coefficient: 0.008 (3)

### 2-Cyano-N'-[(1E)-1-(3,4-dimethoxyphenyl)ethylidene]acetohydrazide Special details

**Table:**

Geometry. All esds (except the esd in the dihedral angle between two l.s. planes) are estimated using the full covariance matrix. The cell esds are taken into account individually in the estimation of esds in distances, angles and torsion angles; correlations between esds in cell parameters are only used when they are defined by crystal symmetry. An approximate (isotropic) treatment of cell esds is used for estimating esds involving l.s. planes.

### 2-Cyano-N'-[(1E)-1-(3,4-dimethoxyphenyl)ethylidene]acetohydrazide Fractional atomic coordinates and isotropic or equivalent isotropic displacement parameters (Å^2^)

**Table:**

	*x*	*y*	*z*	*U*_iso_*/*U*_eq_	Occ. (<1)
O1	0.4532 (2)	0.63384 (18)	−0.0687 (2)	0.0873 (8)	
O2	0.87019 (19)	0.96378 (15)	0.55091 (18)	0.0716 (6)	
O3	1.00787 (18)	0.85504 (16)	0.74705 (17)	0.0680 (6)	
N1	0.3940 (3)	0.9169 (3)	−0.1399 (4)	0.1051 (11)	
N2	0.5860 (2)	0.61306 (19)	0.1263 (2)	0.0626 (7)	
H2	0.585827	0.540243	0.120280	0.075*	
N3	0.65690 (19)	0.66651 (18)	0.2344 (2)	0.0590 (6)	
C1	0.4510 (3)	0.8648 (3)	−0.0567 (3)	0.0750 (9)	
C2	0.5239 (3)	0.8034 (2)	0.0532 (3)	0.0680 (8)	
H2A	0.495556	0.820980	0.132830	0.082*	
H2B	0.608161	0.827932	0.066527	0.082*	
C3	0.5174 (2)	0.6761 (2)	0.0306 (3)	0.0629 (7)	
C4	0.7271 (2)	0.6060 (2)	0.3220 (3)	0.0554 (7)	
C5	0.7404 (3)	0.4793 (2)	0.3148 (3)	0.0791 (9)	
H5A	0.696800	0.452706	0.229983	0.119*	0.5
H5B	0.707704	0.444113	0.383042	0.119*	0.5
H5C	0.825218	0.460173	0.326694	0.119*	0.5
H5D	0.789682	0.451955	0.396497	0.119*	0.5
H5E	0.778777	0.460548	0.243437	0.119*	0.5
H5F	0.661263	0.444489	0.299785	0.119*	0.5
C6	0.8006 (2)	0.6688 (2)	0.4357 (2)	0.0544 (7)	
C7	0.7997 (2)	0.7877 (2)	0.4374 (3)	0.0557 (7)	
H7	0.752355	0.826757	0.366329	0.067*	
C8	0.8673 (2)	0.8482 (2)	0.5422 (2)	0.0544 (7)	
C9	0.9413 (2)	0.7895 (2)	0.6492 (2)	0.0548 (7)	
C10	0.9413 (3)	0.6734 (2)	0.6487 (3)	0.0627 (7)	
H10	0.987959	0.634148	0.720009	0.075*	
C11	0.8724 (3)	0.6137 (2)	0.5426 (3)	0.0646 (8)	
H11	0.874624	0.534744	0.543617	0.078*	
C12	0.8042 (3)	1.0254 (2)	0.4392 (3)	0.0795 (9)	
H12A	0.812865	1.105355	0.456610	0.119*	
H12B	0.836055	1.007186	0.363009	0.119*	
H12C	0.719483	1.005024	0.422915	0.119*	
C13	1.0939 (3)	0.7968 (3)	0.8472 (3)	0.0808 (10)	
H13A	1.135675	0.850615	0.910895	0.121*	
H13B	1.051878	0.742740	0.890291	0.121*	
H13C	1.152076	0.758002	0.807590	0.121*	

### 2-Cyano-N'-[(1E)-1-(3,4-dimethoxyphenyl)ethylidene]acetohydrazide Atomic displacement parameters (Å^2^)

**Table:**

	*U*^11^	*U*^22^	*U*^33^	*U*^12^	*U*^13^	*U*^23^
O1	0.0962 (16)	0.0556 (12)	0.0888 (15)	−0.0086 (11)	−0.0261 (13)	−0.0013 (11)
O2	0.0960 (14)	0.0461 (11)	0.0650 (12)	0.0002 (10)	0.0014 (10)	−0.0039 (9)
O3	0.0821 (13)	0.0586 (11)	0.0568 (11)	−0.0016 (10)	0.0011 (10)	−0.0062 (9)
N1	0.102 (2)	0.081 (2)	0.118 (3)	0.0087 (18)	−0.006 (2)	0.0182 (19)
N2	0.0645 (14)	0.0452 (12)	0.0703 (15)	−0.0035 (10)	−0.0021 (12)	−0.0056 (11)
N3	0.0589 (13)	0.0472 (12)	0.0648 (14)	−0.0062 (10)	0.0006 (11)	−0.0067 (11)
C1	0.0699 (19)	0.0540 (17)	0.095 (2)	0.0030 (15)	0.0049 (18)	0.0030 (17)
C2	0.0673 (17)	0.0479 (15)	0.082 (2)	−0.0003 (13)	0.0028 (15)	−0.0026 (14)
C3	0.0587 (16)	0.0524 (15)	0.0709 (18)	−0.0058 (13)	−0.0004 (14)	−0.0005 (14)
C4	0.0586 (15)	0.0477 (14)	0.0603 (16)	−0.0027 (12)	0.0136 (13)	−0.0004 (12)
C5	0.100 (2)	0.0519 (17)	0.075 (2)	−0.0004 (16)	−0.0025 (17)	−0.0036 (15)
C6	0.0588 (15)	0.0478 (14)	0.0558 (15)	−0.0019 (12)	0.0108 (12)	0.0000 (12)
C7	0.0621 (16)	0.0491 (14)	0.0553 (15)	−0.0007 (12)	0.0113 (13)	0.0010 (12)
C8	0.0614 (15)	0.0480 (14)	0.0534 (15)	−0.0002 (12)	0.0115 (12)	−0.0005 (12)
C9	0.0592 (15)	0.0549 (15)	0.0494 (14)	−0.0010 (12)	0.0103 (12)	−0.0049 (12)
C10	0.0705 (17)	0.0559 (16)	0.0566 (16)	0.0007 (13)	0.0032 (13)	0.0063 (13)
C11	0.0753 (18)	0.0454 (14)	0.0697 (18)	−0.0022 (13)	0.0083 (15)	0.0039 (13)
C12	0.106 (2)	0.0497 (16)	0.073 (2)	−0.0010 (16)	0.0002 (17)	0.0056 (15)
C13	0.085 (2)	0.076 (2)	0.0684 (19)	0.0057 (17)	−0.0101 (16)	−0.0089 (16)

### 2-Cyano-N'-[(1E)-1-(3,4-dimethoxyphenyl)ethylidene]acetohydrazide Geometric parameters (Å, º)

**Table:**

O1—C3	1.225 (3)	C5—H5D	0.9600
O2—C8	1.364 (3)	C5—H5E	0.9600
O2—C12	1.428 (3)	C5—H5F	0.9600
O3—C9	1.360 (3)	C6—C11	1.382 (4)
O3—C13	1.429 (3)	C6—C7	1.401 (4)
N1—C1	1.135 (4)	C7—C8	1.379 (3)
N2—C3	1.339 (3)	C7—H7	0.9300
N2—N3	1.376 (3)	C8—C9	1.412 (3)
N2—H2	0.8600	C9—C10	1.367 (4)
N3—C4	1.280 (3)	C10—C11	1.390 (4)
C1—C2	1.443 (4)	C10—H10	0.9300
C2—C3	1.517 (4)	C11—H11	0.9300
C2—H2A	0.9700	C12—H12A	0.9600
C2—H2B	0.9700	C12—H12B	0.9600
C4—C6	1.480 (3)	C12—H12C	0.9600
C4—C5	1.504 (4)	C13—H13A	0.9600
C5—H5A	0.9600	C13—H13B	0.9600
C5—H5B	0.9600	C13—H13C	0.9600
C5—H5C	0.9600		
			
C8—O2—C12	116.9 (2)	H5A—C5—H5F	56.3
C9—O3—C13	116.3 (2)	H5B—C5—H5F	56.3
C3—N2—N3	119.0 (2)	H5C—C5—H5F	141.1
C3—N2—H2	120.5	H5D—C5—H5F	109.5
N3—N2—H2	120.5	H5E—C5—H5F	109.5
C4—N3—N2	118.6 (2)	C11—C6—C7	117.7 (2)
N1—C1—C2	177.2 (4)	C11—C6—C4	121.9 (2)
C1—C2—C3	112.0 (2)	C7—C6—C4	120.4 (2)
C1—C2—H2A	109.2	C8—C7—C6	121.5 (2)
C3—C2—H2A	109.2	C8—C7—H7	119.2
C1—C2—H2B	109.2	C6—C7—H7	119.2
C3—C2—H2B	109.2	O2—C8—C7	124.7 (2)
H2A—C2—H2B	107.9	O2—C8—C9	115.8 (2)
O1—C3—N2	122.3 (3)	C7—C8—C9	119.5 (2)
O1—C3—C2	122.0 (2)	O3—C9—C10	124.7 (2)
N2—C3—C2	115.7 (2)	O3—C9—C8	116.1 (2)
N3—C4—C6	115.8 (2)	C10—C9—C8	119.2 (2)
N3—C4—C5	124.7 (2)	C9—C10—C11	120.6 (2)
C6—C4—C5	119.5 (2)	C9—C10—H10	119.7
C4—C5—H5A	109.5	C11—C10—H10	119.7
C4—C5—H5B	109.5	C6—C11—C10	121.5 (2)
H5A—C5—H5B	109.5	C6—C11—H11	119.3
C4—C5—H5C	109.5	C10—C11—H11	119.3
H5A—C5—H5C	109.5	O2—C12—H12A	109.5
H5B—C5—H5C	109.5	O2—C12—H12B	109.5
C4—C5—H5D	109.5	H12A—C12—H12B	109.5
H5A—C5—H5D	141.1	O2—C12—H12C	109.5
H5B—C5—H5D	56.3	H12A—C12—H12C	109.5
H5C—C5—H5D	56.3	H12B—C12—H12C	109.5
C4—C5—H5E	109.5	O3—C13—H13A	109.5
H5A—C5—H5E	56.3	O3—C13—H13B	109.5
H5B—C5—H5E	141.1	H13A—C13—H13B	109.5
H5C—C5—H5E	56.3	O3—C13—H13C	109.5
H5D—C5—H5E	109.5	H13A—C13—H13C	109.5
C4—C5—H5F	109.5	H13B—C13—H13C	109.5
			
C3—N2—N3—C4	−176.4 (2)	C12—O2—C8—C9	174.9 (2)
N3—N2—C3—O1	179.3 (3)	C6—C7—C8—O2	−179.6 (3)
N3—N2—C3—C2	−0.9 (4)	C6—C7—C8—C9	1.3 (4)
C1—C2—C3—O1	−2.4 (4)	C13—O3—C9—C10	7.8 (4)
C1—C2—C3—N2	177.8 (2)	C13—O3—C9—C8	−172.6 (2)
N2—N3—C4—C6	−179.9 (2)	O2—C8—C9—O3	−0.9 (3)
N2—N3—C4—C5	1.3 (4)	C7—C8—C9—O3	178.2 (2)
N3—C4—C6—C11	174.5 (3)	O2—C8—C9—C10	178.7 (2)
C5—C4—C6—C11	−6.7 (4)	C7—C8—C9—C10	−2.1 (4)
N3—C4—C6—C7	−5.8 (4)	O3—C9—C10—C11	−178.5 (2)
C5—C4—C6—C7	173.0 (3)	C8—C9—C10—C11	1.9 (4)
C11—C6—C7—C8	−0.4 (4)	C7—C6—C11—C10	0.1 (4)
C4—C6—C7—C8	180.0 (2)	C4—C6—C11—C10	179.8 (2)
C12—O2—C8—C7	−4.2 (4)	C9—C10—C11—C6	−0.9 (4)

### 2-Cyano-N'-[(1E)-1-(3,4-dimethoxyphenyl)ethylidene]acetohydrazide Hydrogen-bond geometry (Å, º)

*Cg* is the centroid of the benzene ring (C6–C11).

**Table:**

*D*—H···*A*	*D*—H	H···*A*	*D*···*A*	*D*—H···*A*
C5—H5*A*···N2	0.96	2.38	2.796 (4)	105
C7—H7···N3	0.93	2.44	2.749 (3)	100
N2—H2···O1^i^	0.86	2.14	2.982 (3)	166
C5—H5*A*···O1^i^	0.96	2.33	3.252 (4)	160
C5—H5*E*···O2^ii^	0.96	2.59	3.436 (4)	147
C13—H13*B*···*Cg*^iii^	0.97	2.86	3.569 (4)	132

Symmetry codes: (i) −*x*+1, −*y*+1, −*z*; (ii) *x*, −*y*+3/2, *z*−1/2; (iii) *x*, −*y*+3/2, *z*+1/2.
